# Structural and Functional Variations of Enzymes Indicate Latitudinal Adaptations of Subarctic and Arctic Sea Urchins

**DOI:** 10.1002/ece3.72199

**Published:** 2025-09-25

**Authors:** Marie Koch, Sylke Wohlrab, Lars Harms, Reinhard Saborowski

**Affiliations:** ^1^ Integrative Ecophysiology Alfred Wegener Institute Helmholtz Centre for Polar and Marine Research Bremerhaven Germany; ^2^ Faculty 2 Biology/Chemistry University of Bremen Bremen Germany; ^3^ Ecological Chemistry Alfred Wegener Institute Helmholtz Centre for Polar and Marine Research Bremerhaven Germany; ^4^ Data Science Support Alfred Wegener Institute Helmholtz Centre for Polar and Marine Research Bremerhaven Germany

**Keywords:** enzyme activities, local adaptation, nutrient utilization, thermal adaptation, transcriptomics

## Abstract

Rapid environmental changes in Arctic fjord systems due to global warming pose new challenges to benthic key species. Sea urchins of the genus *Strongylocentrotus* are the main grazers on habitat‐forming kelp and have significant influence on their environment. Given their ecological relevance, it is crucial to address their potential impacts in future warming systems. We compared sea urchins from the Subarctic Porsangerfjord (Northern Norway) and the Arctic Kongsfjord (Svalbard) to investigate adaptation to local environmental conditions. The two fjords differ in temperature and ice cover. Due to progressive warming, the Arctic Kongsfjord is developing into a glacier‐ and ice‐free fjord, as the Subarctic Porsangerfjord already is. Transcriptomes of two *Strongylocentrotus* species, *S. droebachiensis* and *S. pallidus* , were analyzed for convergent amino acid substitutions. We identified genetic variations in metabolic and energy‐related pathways that may be linked to local thermal environments. Furthermore, we showed that colder‐exposed Arctic sea urchins exhibited higher enzyme activities and lower Arrhenius activation energies, which might be associated with specific amino acid sequence changes. Our results suggest an overall higher biochemical and metabolic efficiency in Arctic sea urchins compared to their Subarctic counterparts, which indicates cold adaptation. The enzymatic properties of sea urchins do not only enhance food utilization under cold conditions but may also increase grazing activity and, thus, grazing pressure on kelp forests in the warming Arctic fjords. This, in turn, could have cascading effects on ecosystem structure and biodiversity in the future Arctic.

## Graphical abstract

Rapid warming in Arctic fjords challenges cold‐adapted benthic species, such as sea urchins of the genus *Strongylocentrotus*, key grazers on kelp forests. By comparing Arctic and Subarctic populations, we identified genetic and enzymatic differences in Arctic sea urchins, suggesting enhanced metabolic efficiency at low temperatures and potential for increased grazing under future warming scenarios.

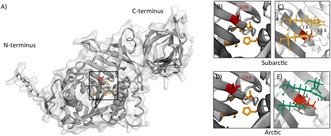

## 1 Introduction

Progressing global warming due to the anthropogenic release of greenhouse gases into the atmosphere affects all terrestrial and marine ecosystems (IPCC 2023). The Arctic, in particular, is subjected to four times higher warming rates than the global average (Rantanen et al. 2022). As a consequence, marine and coastal Arctic ecosystems are experiencing drastic changes in species composition and primary production (Węsławski et al. 2011; Krajewska et al. 2020).

Sea urchins of the genus *Strongylocentrotus* are the key grazers in Subarctic and Arctic fjord systems (Vasseur 1952; Bluhm et al. 1998; Norderhaug et al. 2016; Dvoretsky and Dvoretsky 2024). Through their grazing activities, they significantly influence the distribution and abundance of macroalgae (Hagen 1983; Norderhaug and Christie 2009; Christie et al. 2019). The dominant species *Strongylocentrotus droebachiensis* (O.F. Müller), also known as the green sea urchin, has been responsible for the destruction of around 2000 km^2^ of kelp forests along the Northeast Atlantic coast during the last decades (Norderhaug and Christie 2009). Temperature, one of the main changing abiotic factors, was found to affect the grazing activity of sea urchins (Siikavuoipio et al. 2008; Lawrence et al. 2009; Koch et al. 2024) as well as recruitment and growth (Hart and Scheibling 1988; Pearce et al. 2005; Fagerli et al. 2013). Therefore, the effects of changing conditions in subarctic and arctic ecosystems on sea urchins are of increasing interest in ecology and conservation.

Species respond to changes in their environment either by evasion and relocation, or they undergo physiological and genetic modulations that lead to plastic acclimation or genetic adaptation to mitigate suboptimal living conditions (Franks and Hoffmann 2012; Williams and Blois 2018). Adaptation can occur through small genetic sequence variations, often as single amino acid substitutions, which can modify enzymatic capacities (Somero 2003; Fields et al. 2015). It is well established that, in addition to genetic fluxes, such as those resulting from the dispersal of planktonic larvae, adaptations to local environmental conditions frequently occur among distant populations (Sanford and Kelly 2011). The target species of our study, *S. droebachiensis* and *S. pallidus* , are represented with the North Norwegian populations east of the North Cape and the West‐Svalbard populations, which are over 1000 km apart from each other. *S. droebachiensis* is partially genetically differentiated (Norderhaug et al. 2016).

**FIGURE 1:**
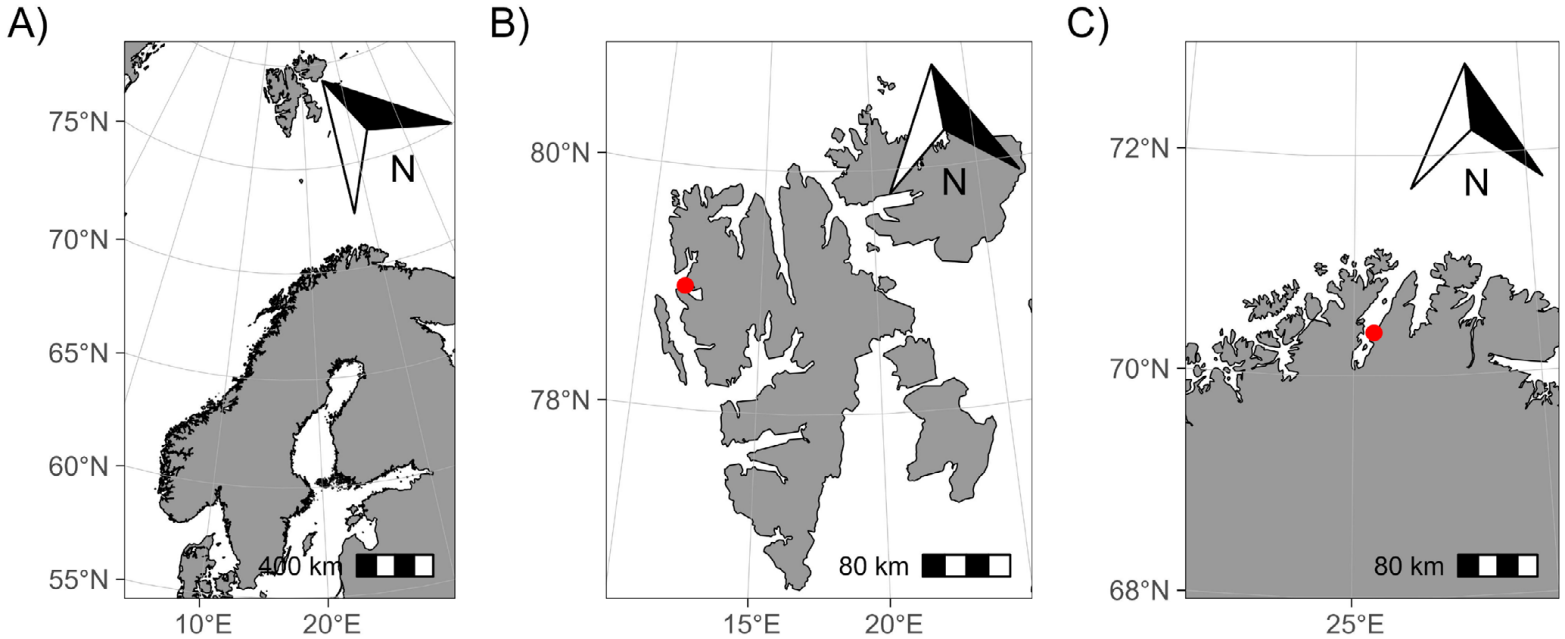
(A) The map depicts Scandinavia and Svalbard were the two sampling sites, (B) Kongsfjord, Svalbard (red dot), and (C) Porsangerfjord, Norway (red dot) are located.

The Arctic Kongsfjord (78°40′ to 77°30′ N, 11°3′ to 13°6′ E, Figure 1A,B) on the west side of the Svalbard Archipelago is shaped by several sea‐terminating glaciers. It shows a mean winter temperature of approximately −2°C and mean summer temperatures up to 6°C (Richard et al. 2012; Aliani et al. 2016; Noufal et al. 2017). The temperature of the fjord is vastly influenced by warm Atlantic water inflow from the mouth of the fjord and glacial meltwater inflow at the inner part of the fjord (Hegseth and Tverberg 2013; Sundfjord et al. 2017). In contrast, the Subarctic North Norwegian Porsangerfjord (70.0°–71.0° N, 25.0°–26.5° E, Figure 1A,C) is a glacier‐free fjord with a temperature range from 2.5°C in March to 10°C in August (Koch et al. 2024). It has been proposed that glacial‐free Subarctic fjord systems, such as the Porsangerfjord, could serve as a template to study and understand the future development of high Arctic fjord ecosystems under continuous warming (Balazy and Kuklinski 2019).

The aim of this study was to assess potential thermal adaptations of the benthic key grazers *S. droebachiensis* and *S. pallidus* . We investigated transcriptomic variations and differences in enzyme activities between Subarctic and Arctic sea urchin populations to determine the plasticity of these key grazers in relation to the temperature regime. While the effects of temperature on behavior, growth, and physiology of these grazers have been the subject of several previous studies, the plasticity of their enzymes in relation to temperature has not been investigated so far. We focused on variations of enzyme activities at environmental temperatures as well as the identification of so‐called convergent amino acid substitutions (CAAS). Convergent evolution describes the emergence of certain phenotypes in independent groups that are subjected to different environmental conditions. It can be identified by the comparison of amino acid sequences (Rey et al. 2019). We proposed genetic differences in the populations from two varying habitats that can be related to local environmental conditions and are reflected in the kinetic properties of enzymes, important in energy uptake and digestion. Based on these findings, we hypothesized about the fate of these key grazers in future warming fjords.

## 2 Material and Methods

### 2.1 Samples

Individuals of *S. droebachiensis* and *S. pallidus* were collected at 1‐m depth in the Porsangerfjord, Northern Norway (70°22′ N, 25°28′ E, Figure 1B) in July 2022 and August 2023 and by SCUBA divers at 2–8‐m depth in March 2023 and in May 2024 in Kongsfjord, Svalbard (78°55′ N, 11°54′–11°56′ E, Figure 1C). Sea urchins were dissected in the respective local laboratory. Stomach and intestine tissue were rinsed with distilled water. Samples for enzyme activity assays were shock frozen in liquid nitrogen. Tissue samples for RNA extraction were preserved in RNAlater (Invitrogen, Cat. no.: AM7020). To ensure optimum RNA preservation, samples were first kept in RNAlater at room temperature for 24 h and then frozen at −80°C. The frozen samples were transported in a dry shipper (−198°C) to the laboratory facilities of the Alfred‐Wegener‐Institute, Germany, and stored at −80°C until further analysis.

### 2.2 RNA Isolation and Sequencing

RNA was isolated from approximately 20 mg stabilized stomach and intestinal tissue of *S. droebachiensis* and *S. pallidus* originating from both sampling locations (*n* = 3, each). Additionally, RNA from the genetically more detailed analyzed purple sea urchin *Strongylocentrotus purpuratus* (*n* = 3) was extracted, as the transcriptome was used for later orthogroup identification (see Section 2.4). Tissue disruption was carried out in 0.6 mL RLT buffer using a Precellys homogenizer (Bertin Technologies, Montigny‐Bretonneux, France) at 6000 rpm for 16 s. RNA purification was performed with the RNeasy mini kit (Qiagen, Cat. no.: 74104) as per the producer's instructions. RNA quantity and purity were checked by Nanodrop photometry (Nanodrop spectrometer, ND‐1000) at 260/230 nm and 260/280 nm, respectively. RNA from both types of tissue from the same sampling location was combined to form one pooled sample per location and species with the same RNA concentration of each tissue and from each sample. RNA library preparation and sequencing were carried out by the company AllGenetics & Biology SL (www.allgenetics.eu) on an Illumina PE150 Sequencing platform (approx. 200 Mio. reads). Raw sequencing reads were received from the company after de‐multiplexing and adapter‐cleaning.

### 2.3 De Novo Transcriptome Assembly

Paired‐end read files were preprocessed as follows: adapter sequences were removed using BBDuk v39.01 (Bushnell, B. https://sourceforge.net/projects/bbmap/) with the settings ktrim = *r*, k23, mink = 11, hdist = 1, tpe, and tbo. rRNA sequences were then removed using SortMeRNA v4.3.6 (Kopylova et al. 2012) with default parameters. Subsequently, Illumina Phix standards were removed, and right‐end quality trimming (qtrim = *r*, trimq = 10, minlen = 36) was performed with BBDuk. The quality of the reads was checked with FastQC (Andrews 2010).

De novo transcriptome assembly was performed using Trinity v2.9.1 with the standard pipeline (Grabherr et al. 2013). Functional annotation was conducted with Trinotate v4.0.1 (Bryant et al. 2017), including alignment of transcripts to the UniProt protein database (accessed December 31, 2023) and identification of likely coding regions using TransDecoder v5.7.0 (Haas, BJ. https://github.com/TransDecoder/TransDecoder). Potential transmembrane regions of the resulting proteins were predicted using TMHMM v2.0c (Krogh et al. 2001), and signal peptides were detected using SignalP v6.0g (Teufel et al. 2022). Orthologous groups were assigned using eggNOG‐mapper v2.1.12 (Cantalapiedra et al. 2021). All annotation results were compiled into an SQLite database and exported as a comprehensive transcript annotation file.

The completeness of transcriptomes was assessed using BUSCO v5.7.4 (Manni et al. 2021) against the eukaryote_odb10 database, which includes 255 conserved single‐copy orthologs. To evaluate the assembly representation, paired‐end reads were aligned back to the assembly using Bowtie2 (Langmead and Salzberg 2012), and the proportion of concordant alignments was calculated. Assembly contiguity was assessed by calculating N50 with Trinity's built‐in utility scripts, following the recommended guidelines (Haas BJ, https://github.com/trinityrnaseq/trinityrnaseq/wiki/Transcriptome‐Contig‐Nx‐and‐ExN50‐stats).

### 2.4 Detection and Analysis of Convergent Amino Acid Substitutions

The transcriptomes were screened for potential CAAS using CAAStools (Barteri et al. 2023) and the TransDecoder output files from the Trinity assembly. First, orthologs were identified with OrthoFinder v2.5.5 (Emms and Kelly 2019), including the transcriptome of *S. purpuratus* for more adequate orthologous gene identification. However, this transcriptome was not considered further for identifying CAAS between the two populations of *S. droebachiensis* and *S. pallidus* . Each cluster was individually aligned using MAFFT v7 (Katoh et al. 2002), and the alignments were trimmed using the “gappyout” algorithm implemented in TrimAl v1.2rev59 (Capella‐Gutiérrez et al. 2009) to remove poorly aligned and gap‐rich regions. For the detection of amino acid substitutions, transcriptomes of *S. droebachiensis* and *S. pallidus* from the Kongsfjord were designated as the foreground group. CAAStools was run using the ct module, and the results were stored in output files for downstream analysis.

Clusters containing potential CAAS were functionally annotated using eggNOG‐mapper v2.1.12 with an *e*‐value threshold of 0.001 and a minimum query coverage of 20%. The resulting annotations included eggNOG orthologous groups, COG functional categories, Gene Ontology (GO) terms, KEGG pathway assignments, CAZy classifications, and Enzyme Commission (EC) numbers. Multiple sequence alignments of orthogroups containing candidate CAAS were manually inspected, and orthogroups were excluded from further analysis if alignment quality was poor or if substitutions occurred in highly variable regions.

The amino acid flux (*D*) between locations was calculated following the method described by Yampolsky and Bouzinier (2010), which quantifies the net relative gain or loss of amino acids through substitutions:

$$D=\frac{C-R}{C+R}$$

*C* represents the number of events an amino acid was gained (created) in the Svalbard populations, whereas *R* indicates how often the same amino acid was lost (removed) in the same population.

### 2.5 Enzyme Extraction

Crude extracts of stomach and intestine tissue (*n* = 5) from *S. droebachiensis* from both locations were prepared by homogenizing the frozen tissue with a micro‐pestle in a 2‐mL reaction cup. Weighted tissue was processed on ice for 15 s in extraction buffer (0.1 mol L^−1^ Tris–HCl, 0.1 mmol L^−1^ EDTA, 0.1% Triton X‐100, ratio 1:5, w/v). The homogenates were centrifuged at 25,000 relative centrifugal force (rcf) for 30 min at 4°C. The supernatant was transferred into new cups and again centrifuged at 15,000 rcf for 10 min at 4°C to remove any remaining impurities. The supernatant was stored at −80°C until further analysis.

### 2.6 Temperature Profiles of Enzyme

Digestive enzyme activities were measured at 2°C, 5°C, 10°C, 15°C, 20°C, 30°C, 40°C, and 50°C using colorimetric and fluorogenic substrates (Table 1) as described by Koch et al. (2025). In short, for the fluorometric assays, 10 μL of enzyme extract was added to 990 μL of pre‐tempered assay buffer, containing 0.1 mmol L^−1^ substrate dissolved in DMSO. After mixing, the increase in fluorescence (*λ*_ex_ = 355 nm and *λ*_em_ = 469 nm) was recorded over 5 min with a temperature‐controlled spectrofluorometer (FP‐8250ST; Jasco Global). Colorimetric assays were performed in a temperature‐controlled spectrophotometer (Specord 200 plus; AnalytikJena, Jena, Germany). Twenty‐five microliter of enzyme extract was added and mixed with 465 μL of assay buffer and adjusted to the assay temperature. To start the reaction, 10 μL of 50 mmol L^−1^ of substrate dissolved in DMSO was added, and the increase in absorbance at 405 nm was measured over 5 min. Enzyme activities were calculated as detailed by Koch et al. (2025).

**TABLE 1:** Substrates used for enzyme activity assays.

Substrate	Source	Cat. no.	Representing enzyme	Assay buffer
**Fluorogenic assays**				
MUF‐butyrate	Fluka	19362	Esterase (C4)	0.1 mol L^−1^ Na‐Phos (pH 7)
MUF‐heptanoate	Sigma	M2514	Esterase/Lipase (C7)	
MUF‐α‐d‐glucopyranoside	Fluka	96591	α‐glucosidase	
MUF‐β‐d‐glucopyranoside	Sigma	M3633	β‐glucosidase	
MUF‐β‐d‐galactoside	Sigma	M1633	β‐galactosidase	
**Colorimetric assays**				
l‐Leucine p‐nitroanilide hydrochloride	Sigma	L2158	Leucin‐aminopeptidases	0.1 mol L^−1^ Tris–HCl (pH 8)
l‐Alanine p‐nitroanilide hydrochloride	Sigma	A9325	Alanine‐aminopeptidases	

Abbreviation: MUF, 4‐methylumbelliferone.

### 2.7 Activation Energy *E*_a_

In order to compare the energetic properties of digestive enzymes from *S. droebachiensis* from two different populations, the activation energy *E*_a_ was calculated. *E*_a_ describes the energy barrier that must be overcome for a chemical reaction to take place. *E*_a_ can be calculated from the Arrhenius equation as follows:

$$k={\mathrm{Ae}}^{-E_{a}/\left(\mathrm{RT}\right)}$$

with *k* representing the reaction rate, *R* the universal gas constant, *T* the absolute temperature, and *A* the pre‐exponential factor. *E*_a_ was determined from the linearized Arrhenius plot.

### 2.8 Statistics

All statistical analyses were conducted with R v4.4.2 (R Core Team 2024) if not otherwise stated. Tissue‐specific enzyme activity at given temperatures was tested for normal distribution and variance homogeneity. Subsequently, a one‐way analysis of variance (ANOVA) followed by a multiple comparison of means (Tukey's HSD) was conducted.

### 2.9 Protein Structure Models and Stability Calculation

Protein structures based on sequences of TAG‐lipase, alanine‐aminopeptidase, and β‐galactosidases in which CAAS were modeled using SWISS‐Model (https://swissmodel.expasy.org/). For this, the automated mode and the resulting top‐ranked templates were used. Further visualization of the models was conducted with the EzMol interface (Reynolds et al. 2018). The effect of the detected substitutions on the enzyme stability was evaluated using a Cutoff Scanning Matrix (mCSM, https://biosig.lab.uq.edu.au/mcsm/stability) approach, which allows to calculate the change in ΔΔ*G* (Pires et al. 2014). Exemplary contigs of each species were used for the calculation of the ΔΔ*G* change caused by the single amino acid substitution. Protein weight derived from amino acid sequences was calculated using the Protein Molecular Weight Calculator from The Sequence Manipulation Suite (https://www.bioinformatics.org/sms/index.html). Putative catalytic residues were derived from the protein structure models using the software PDBSiteScan (Ivanisenko et al. 2004). Hydrogen bonds were detected using ProteinTools (https://proteintools.uni‐bayreuth.de/; Ferruz et al. 2021).

## 3 Results

### 3.1 De Novo Transcriptome Assembly

**FIGURE 2:**
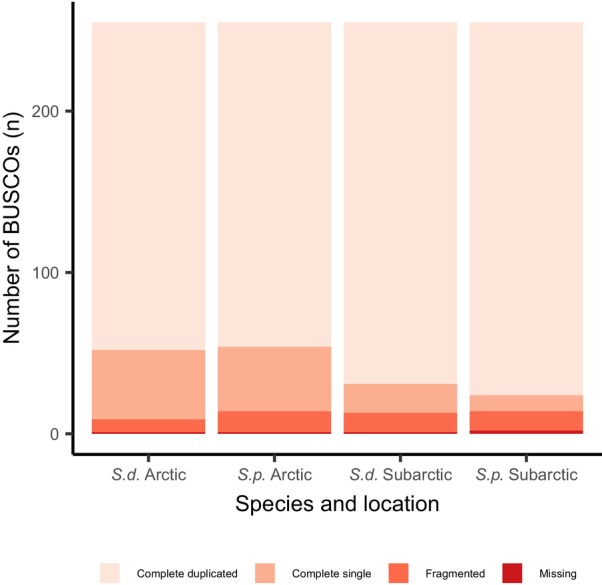
The completeness of the transcriptomes of *Strongylocentrotus droebachiensis* (*S.d*.) and *Strongylocentrotus pallidus* (*S.p*.) assessed by searching for benchmark universal single‐copy orthologs (BUSCOs) using the reference set for eukaryotes.

**FIGURE 3:**
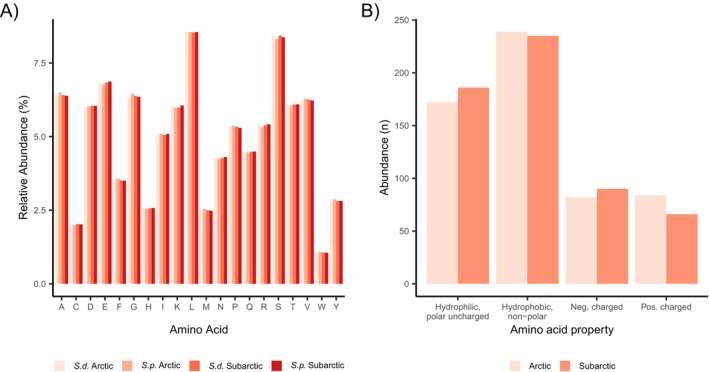
(A) Amino acid composition in the likely coding regions of the four transcriptome assemblies of *Strongylocentrotus droebachiensis* (*S.d*.) and *Strongylocentrotus pallidus* (*S.p*.). (B) Distribution of hydrophilic (N, Q, S, T, Y), hydrophobic (A, C, F, G, I, L, M, P, V, W), negatively (D, E), and positively (H, K, R) charged amino acid residues involved in CAAS in Subarctic and Arctic sea urchins.

RNAseq data for *S. droebachiensis* and *S. pallidus* from the Kongsfjord and the Porsangerfjord were assembled de novo into four resulting transcriptomes. The overall alignment rate was 98%, with no more than one BUSCO missing per transcriptome in all four transcriptomes (Table 1, 2, Figure 2). Each assembly contained a minimum of 396,000 transcripts, grouped into at least 223,000 contigs. The mean contig length of the transcriptomes was between 688 and 829 base pairs (bp). The amino acid composition was uniform across all four transcriptomes, with leucine and serine being the most prevalent amino acids (Figure 3A).

**TABLE 2:** De novo transcriptome assembly statistics for the four transcripts of Subarctic and Arctic sea urchins.

	*S. droebachiensis*		*S. pallidus*		*S. purpuratus*
	Porsangerfjord	Kongsfjord	Porsangerfjord	Kongsfjord	Commercial
No. of sampled individuals	3	3	3	3	3
No. of sampled tissue	2	2	2	2	2
Total pair of paired‐end reads	104,054,994	109,439,962	89,987,385	100,585,798	97,217,202
Total trinity transcripts	548,689	396,457	563,818	526,908	464,440
Total trinity “genes”	303,189	223,464	276,424	269,971	250,444
Percent GC	39.34	39.67	39.51	39.59	39.10
Contig N50 (bp)	1191	1528	1070	1014	1256
Median contig length (bp)	389	409	403	399	429
Mean contig length (bp)	729.40	828.61	710.07	687.62	776.70
Total assembled bases	400,211,920	328,506,798	400,347,803	362,313,359	360,732,158
Overall alignment rate	98.00%	98.33%	97.92%	98.36%	98.56%
Genes in orthogroups	104,150	84,581	112,437	104,449	94,469

### 3.2 Convergent Amino Acid Substitutions

A total of 576 CAAS were identified between populations from the two fjords (Table 3). Based on the Clusters of Orthologous Groups (COG) categorization, the majority of CAAS were found in transcripts involved in cellular processes and signaling (37.6%), followed by those associated with metabolism (22.9%) and information storage and processing (10.2%). A substantial proportion (29.3%) of proteins was assigned as poorly characterized.

**TABLE 3:** The number and distribution of convergent amino acid substitution (CAAS) occurring between Subarctic and Arctic populations according to the eggNOG categorization. The distribution of CAAS among subcategories is given in % within the main category (%_cat_) as well as % of all occurring CAAS (%_all_).

Category	Subcategory (letter code)	Count	%_cat_	%_all_
Cellular processes and signaling	Signal transduction mechanisms (T)	78	35.94	13.52
	Posttranslational modification, protein turnover, chaperones (O)	64	29.49	11.09
	RNA processing and modification (A)	19	8.76	3.29
	Intracellular trafficking, secretion, and vesicular transport (U)	14	6.45	2.43
	Cytoskeleton (Z)	11	5.07	1.91
	Chromatin structure and dynamics (B)	9	4.15	1.56
	Defense mechanisms (V)	9	4.15	1.56
	Cell cycle control, cell division, chromosome partitioning (D)	6	2.76	1.04
	Cell wall/membrane/envelope biogenesis (M)	3	1.38	0.52
	Extracellular structures (W)	3	1.38	0.52
	Nuclear structure (Y)	1	0.46	0.17
Metabolism	Carbohydrate transport metabolism (G)	34	25.76	5.89
	Lipid transport metabolism (I)	29	21.97	5.03
	Amino acid transport metabolism (E)	26	19.7	4.51
	Energy production conversion (C)	15	11.36	2.6
	Secondary metabolites biosynthesis transport catabolism (Q)	13	9.85	2.25
	Inorganic ion transport metabolism (P)	9	6.82	1.56
	Nucleotide transport metabolism (F)	6	4.55	1.04
Information storage and processing	Translation, ribosomal structure and biogenesis (J)	23	38.98	3.99
	Transcription (K)	18	30.51	3.12
	Replication, recombination, and repair (L)	16	27.12	2.77
	RNA processing and modification (A)	2	3.39	0.35
Poorly characterized	Function unknown (S)	169	100	29.29

**FIGURE 4:**
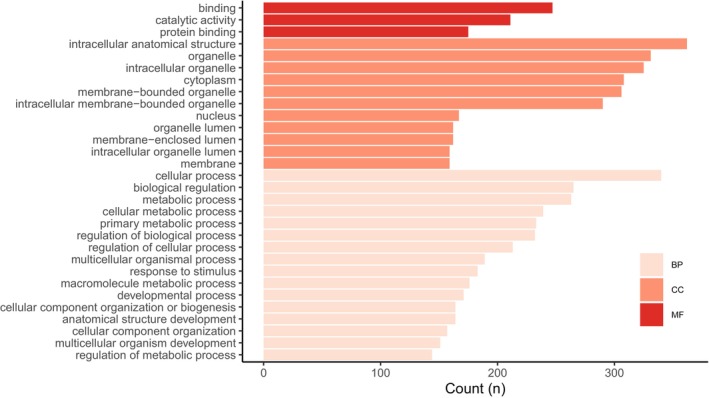
The bar plot depicts the 30 most frequently GO terms associated with contigs, in which CAAS were detected. GO terms are categorized into biological processes (BP), cellular components (CC), and molecular functions (MF).

Most transcripts containing CAAS were annotated with Gene Ontology (GO) terms related to biological processes (BP), such as primary metabolic processes and biological regulation (Figure 4). CAAS‐containing proteins associated with cellular components (CC) were predominantly linked to intracellular anatomical structures and organelles. GO terms describing molecular functions (MF) were less frequently assigned.

Several CAAS occurred in enzymes involved in carbohydrate, lipid, and protein metabolism, including triacylglycerol‐lipase (threonine to serine), metalloaminopeptidase (aspartic acid to glutamic acid), and β‐galactosidase (isoleucine in Kongsfjord population to valine in Porsangerfjord population). One substitution, arginine (Kongsfjord population) to serine (Porsangerfjord population), was detected in the pyruvate‐dehydrogenase complex as well as in malate‐dehydrogenase, where glutamine (Kongsfjord population) was exchanged for asparagine (Porsangerfjord population). Both enzymes are involved in the energy metabolism. A comprehensive list of all identified CAAS, including the number of associated contigs per transcriptome and functional annotation, is provided in Table S1.

**FIGURE 5:**
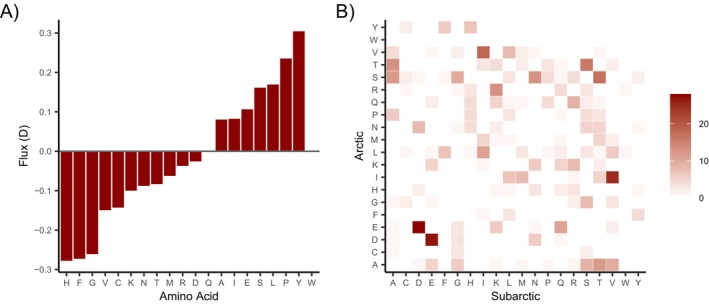
(A) Amino acid flux between Subarctic and Arctic sea urchins. (B) Number of exchanges between amino acids in Subarctic and Arctic sea urchins.

Amino acid substitutions resulted in a higher number of hydrophilic and negatively charged residues, but fewer hydrophobic as well as positively charged amino acids in the Arctic population compared to the Subarctic populations (Figure 3B). In the Kongsfjord populations, the most frequently lost amino acid was tyrosine (~10%). However, the highest negative flux, indicating higher numbers of loss events than new occurrences due to mutation, occurred in histidine (~4%), followed by phenylalanine and glycine. The most frequently gained amino acids were tyrosine, proline, leucine, and serine (Figure 5A). Histidine, a positively charged, polar amino acid, was mostly replaced by the hydrophilic (polar) asparagine and tyrosine. The nonpolar amino acid phenylalanine was mostly replaced by the smaller amino acid leucine and the polar tyrosine. Nonpolar glycine showed high replacement rates with serine, a polar amino acid. The most common substitution was the replacement of asparagine with glutamine (Figure 5B). The arginine/lysine ratio, a common indicator for cold adaptation, was slightly lower in the Arctic populations ( *S. droebachiensis* : 0.934, *S. pallidus* : 0.928) than in the Subarctic populations ( *S. droebachiensis* : 0.944, *S. pallidus* : 0.947).

### 3.3 Enzyme Activities

**FIGURE 6:**
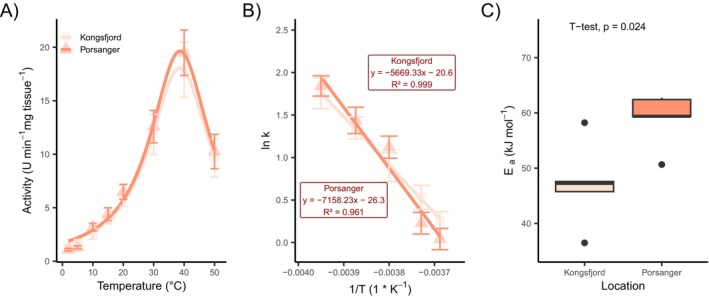
(A) Temperature curve of esterase (C4) from the intestine tissue of *Strongylocentrotus droebachiensis* . The mean and the standard error are shown as points for both populations. (B) Linearized Arrhenius plot derived from temperature data between 2°C and 20°C and (C) the activation energy. The boxplots represent data (*n* = 5) within the first and third interquartile range (IQR) and the median marked as a horizontal line. Points outside of the boxplot show data outside the 90% IQR.

**FIGURE 7:**
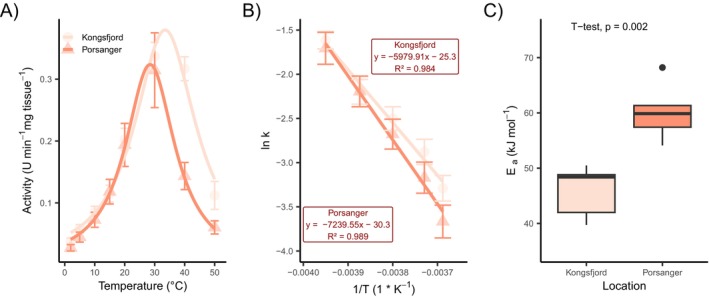
(A) Temperature curve of alanine‐aminopeptidase from the intestine tissue of *Strongylocentrotus droebachiensis* . The mean and the standard error are shown as points for both populations. (B) Linearized Arrhenius plot derived from temperature data between 2°C and 20°C and (C) the consequently calculated activation energy. The boxplots represent data (*n* = 5) within the first and third interquartile range (IQR), with the median being marked as a horizontal line in the middle. Points outside of the boxplot show data outside the 90% IQR.

**FIGURE 8:**
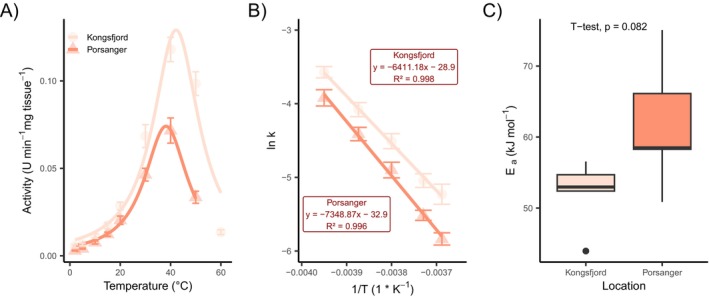
(A) Temperature curve of β‐galactosidase in stomach tissue of *Strongylocentrotus droebachiensis* . The mean and the standard error are shown as points for both populations. (B) Linearized Arrhenius plot derived from temperature data between 2°C and 20°C and (C) the consequently calculated activation energy. The boxplots represent data (*n* = 5) within the first and third interquartile range (IQR), with the median being marked as a horizontal line in the middle. Points outside of the boxplot show data outside the 90% IQR.

The activities of selected digestive enzymes from the stomach and the intestine of urchins, *S. droebachiensis*, were measured within a temperature range of 2°C–50°C (Figures 6A, 7A, and 8A). Enzyme activities in the Kongsfjord population were significantly higher than those of the Porsangerfjord population at environmental summer temperatures of the sampling locations (5°C and 10°C, respectively) for alanine‐aminopeptidase, leucine‐aminopeptidase, β‐galactosidase (Figure 8A), and β‐glucosidase in the stomach. Although these enzymes also exhibit slightly elevated activity in the intestine, the differences between populations were not statistically significant (Figure 7A, Table 4).

**TABLE 4:** Mean and standard deviation of enzyme activities in the stomach and intestine of *S. droebachiensis* from the Arctic and the Subarctic at 5°C and 10°C, as well as activation energy *E*_a_, calculated from enzyme activity data between 2°C and 20°C. *p* values (significant when < 0.05) are given for *t*‐tests between data from Arctic and Subarctic populations.

Tissue	Enzyme	5°C			10°C			*E*_a_		
		Arctic	Subarctic	*p*	Arctic	Subarctic	*p*	Arctic	Subarctic	*p*
Stomach	Esterase (C4)	2.404 ± 0.189	2.038 ± 0.688	0.21	3.565 ± 0.519	4.901 ± 1.520	0.12	50.102 ± 7.144	60.406 ± 7.658	0.09
	Esterase (C7)	3.895 ± 0.654	4.322 ± 1.416	0.56	4.642 ± 0.644	7.113 ± 2.121	0.06	51.017 ± 5.672	53.396 ± 2.941	0.44
	Ala‐AP	0.045 ± 0.009	0.031 ± 0.009	0.04	0.067 ± 0.013	0.042 ± 0.011	0.01	49.579 ± 2.460	47.642 ± 7.757	0.62
	Leu‐AP	0.032 ± 0.010	0.013 ± 0.003	0.01	0.043 ± 0.018	0.022 ± 0.005	0.06	43.621 ± 5.838	45.490 ± 7.923	0.68
	α‐glu	0.033 ± 0.003	0.033 ± 0.014	0.96	0.053 ± 0.006	0.049 ± 0.024	0.72	55.964 ± 3.617	43.840 ± 5.693	0.01
	β‐gala	0.007 ± 0.001	0.004 ± 0.001	0.01	0.011 ± 0.003	0.008 ± 0.002	0.05	52.109 ± 4.843	61.766 ± 9.190	0.08
	β‐glu	0.044 ± 0.014	0.018 ± 0.004	0.01	0.062 ± 0.022	0.027 ± 0.007	0.02	48.319 ± 5.036	41.935 ± 4.911	0.08
Intestine	Esterase (C4)	1.773 ± 0.558	1.294 ± 0.338	0.15	2.588 ± 1.178	3.171 ± 0.843	0.40	47.058 ± 7.724	58.907 ± 4.888	0.02
	Esterase (C7)	2.568 ± 1.075	1.918 ± 0.513	0.27	4.568 ± 2.131	2.862 ± 0.654	0.15	51.852 ± 6.706	55.063 ± 3.029	0.37
	Ala‐AP	0.058 ± 0.015	0.045 ± 0.018	0.23	0.085 ± 0.022	0.073 ± 0.027	0.47	45.881 ± 4.725	60.190 ± 5.255	0.002
	Leu‐AP	0.033 ± 0.012	0.031 ± 0.008	0.74	0.047 ± 0.018	0.052 ± 0.009	0.56	39.255 ± 3.903	55.345 ± 9.528	0.02
	α‐glu	0.032 ± 0.004	0.031 ± 0.014	0.88	0.051 ± 0.006	0.048 ± 0.023	0.80	51.171 ± 3.468	54.148 ± 3.722	0.23
	β‐gala	0.006 ± 0.001	0.006 ± 0.002	0.51	0.013 ± 0.004	0.009 ± 0.003	0.12	52.046 ± 5.866	47.357 ± 5.032	0.21
	β‐glu	0.090 ± 0.058	0.040 ± 0.011	0.13	0.134 ± 0.076	0.059 ± 0.016	0.09	41.962 ± 4.502	40.540 ± 4.496	0.63

Esterase (C4) and esterase/lipase (C7) activities in the stomach tissue were higher in the Kongsfjord population at 10°C, but not at 5°C (Figure 6A). No significant differences in α‐glucosidase activity were observed between the populations at either temperature (Table 4).

Temperature sensitivity of enzyme activity was assessed using linearized Arrhenius plots, from which activation energies (*E*_a_) were calculated. The Porsangerfjord population showed significantly higher *E*_a_ values for alanine‐aminopeptidase (Figure 7B,C), leucine‐aminopeptidase, and esterase (Figure 6B,C) in the intestine. In the stomach, esterase and β‐galactosidase (Figure 8B,C) also showed elevated *E*_a_ values in the Porsangerfjord population, although these differences were not always statistically significant. In contrast, α‐glucosidase exhibits a significantly higher *E*_a_ in the stomach of the Kongsfjord population (Table 4).

### 3.4 Structural Investigation of Selected Digestive Enzymes

Two of the seven biochemically characterized digestive enzymes included CAAS, namely alanine‐aminopeptidase (named metallopeptidase in the CAAS analysis) and β‐galactosidase. Furthermore, additionally one substitution occurred in triacylglycerol (TAG)‐lipase, an esterase involved in lipid degradation. All relevant transcripts were further verified by BLASTP (Standard databases) for correct annotation (Tables S2–S4).

**FIGURE 9:**
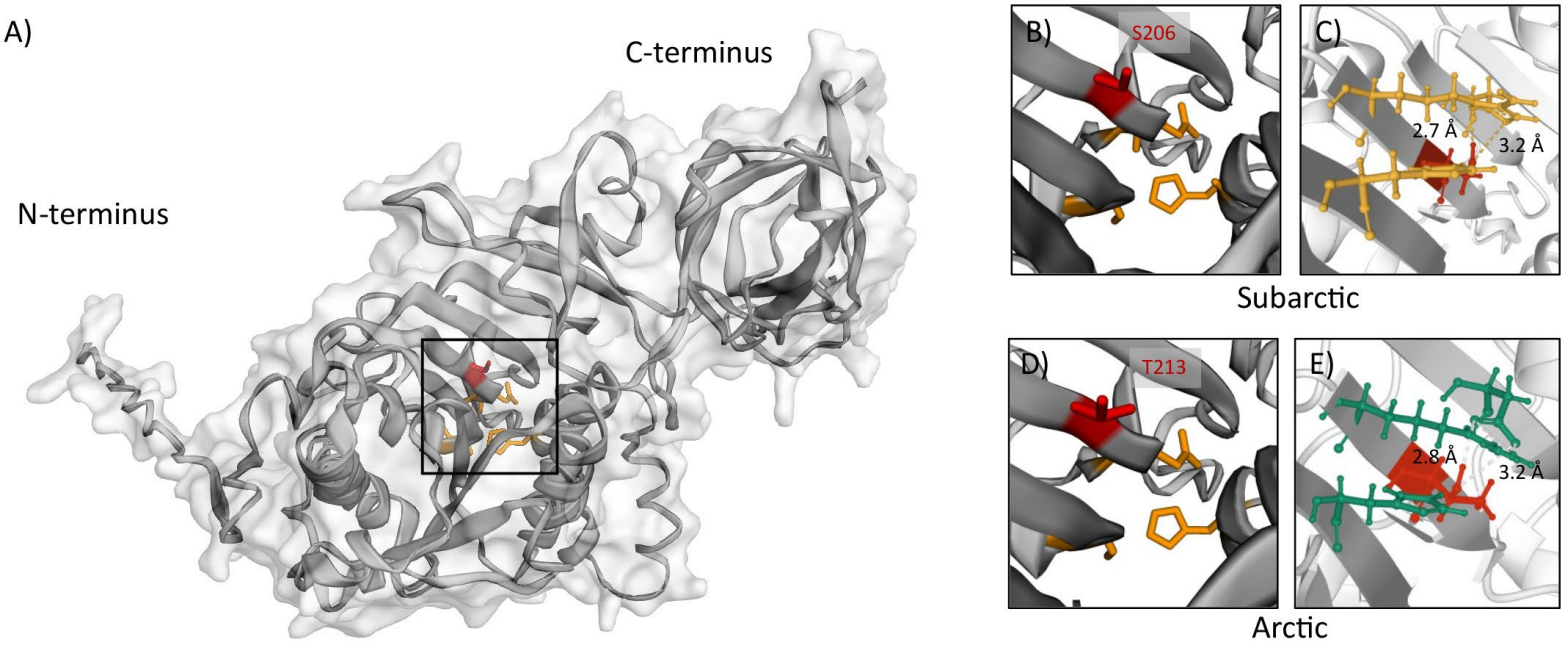
(A) Computational model of a TAG‐lipase of *Strongylocentrotus droebachiensis* . The CAAS side is highlighted in red, the residues of the catalytic triad (histidine, serine, and aspartic acid) are highlighted in orange. (B) The serine residue in the Subarctic population was replaced by (D) threonine in the Arctic enzyme. (C) The Subarctic serine (red) was part of two hydrogen bonds, which are marked as yellow dashed lines, amino acids of this hydrogen bond network are highlighted in yellow as well. (E) The threonine residue (red) of the Arctic enzyme was part of two hydrogen bonds (network residues marked in green).

In total, 14 TAG‐lipase transcripts with amino acid substitutions between the Subarctic and Arctic populations of both *Strongylocentrotus* species were identified. Arctic *S. pallidus* showed the highest number of isoenzymes with six transcripts. The transcripts length was between 492 and 508 amino acids, corresponding to enzymes with molecular masses between 55.4 and 57.2 kDa. Protein structures were modeled after the AlphaFold template A0A7M7TG20 (lipase domain containing protein, *S. purpuratus* ) (Figure 9A–E). The sequence identity coverage was at least 96%.

In the four transcriptomes, a CAAS was found in 15 transcripts annotated as alanine‐aminopeptidase. A glutamic acid was present in the Subarctic populations, while an aspartic acid appeared in the Arctic populations. The transcript sequences were between 625 and 1037 amino acids long, with molecular masses between 72.1 and 118.2 kDa. Enzyme molecule models were successfully calculated using the AlphaFold template A0A7M7HHI0 of an aminopeptidase from *S. droebachiensis* with a sequence identity coverage of at least 92.5%.

Within the four transcriptomes, a total of 17 transcripts identified as β‐galactosidase showed a relevant amino acid substitution, with the *S. droebachiensis* population from the Arctic carrying the highest isoform number of six transcripts. The sequence length was 662 amino acids, resulting in a molecular mass of 75.4 kDa. The CAAS is located at position 61 on the surface of the enzyme. β‐galactosidase models were constructed after the AlphaFold template A0A7M7NCE0 (β‐galactosidase, *S. purpuratus* ), with a sequence identity coverage of at least 98%.

Changes in ΔΔ*G* were calculated for transcripts from both species by simulating reciprocal point mutations of the exchanged amino acid, that is, the one occurring in the Arctic population into the Subarctic transcripts, and vice versa. Destabilizing effects for all analyzed point mutations were found, regardless of population (Table 5). However, the destabilizing effect of the simulated mutation in β‐galactosidase and TAG‐lipase was notably higher in the Arctic populations than in the Subarctic populations.

**TABLE 5:** Overview of ΔΔ*G* of simulated point mutations, number, and position of hydrogen bonds. Negative ΔΔ*G* values indicate destabilizing effects, positive values stabilizing effects. *N* is the number of hydrogen bonds, the number in brackets shows the number of hydrogen bond networks. No bonds indicate no involvement of amino acid from the substitutions site in hydrogen bonds.

Location	Wild‐type contig	Substitution	ΔΔ*G* (kcal/mol)	Hydrogen bonds		
				*N*	Donor—Acceptor	Distance (Å)
**TAG‐lipase**						
*Porsangerfjord*	*S. pallidus* _0052257	S201T	−0.66	62 (35)	H176—S201	2.7; 3.2
					S201—D228	
*Kongsfjord*	*S. pallidus* _0029448	T213S	−1.918	57 (31)	H188—T213	2.8; 3.2
					T213—D240	
*Porsangerfjord*	*S. droebachiensis* _0022207	S206T	−0.731	62 (36)	H181—S206 S206—D233	2.7; 3.2
*Kongsfjord*	*S. droebachiensis* _0001080	T213S	−1.918	62 (35)	H188—T213 T213—D240	2.8; 3.2
**Alanine‐aminopeptidase**						
*Porsangerfjord*	*S. pallidus* _0096851	E465D	−0.798	107 (67)	No bonds	
*Kongsfjord*	*S. pallidus* _0025443	D273E	−0.483	78 (49)	No bonds	
*Porsangerfjord*	*S. droebachiensis_*0072654	E628D	−1.277	118 (69)	N681—E628	2.8
*Kongsfjord*	*S. droebachiensis* _0008839	D667E	−0.525	133 (79)	T613—D667	2.7
**β‐galactosidase**						
*Porsangerfjord*	*S. droebachiensis* _0069224	V61I	−0.71	92 (49)	No bonds	
*Kongsfjord*	*S. droebachiensis_*0050921	I61V	−1.472	98 (50)	No bonds	
*Porsangerfjord*	*S. pallidus_*0103753	V61I	−0.704	98 (50)	No bonds	
*Kongsfjord*	*S. pallidus* _0049908	I61V	−1.475	92 (50)	No bonds	

The involvement of the investigated amino acid residues in hydrogen bond networks was analyzed (Table 5). Neither V61 nor I61 in the β‐galactosidase transcript was involved in hydrogen bond networks. Both enzymes, TAG‐lipase and alanine‐aminopeptidase, showed only small changes (max. 0.1 Å) in residue distance at the substitution site between locations. In TAG‐lipase, the distance was smaller in the Subarctic populations than in the Arctic populations. The opposite was true for alanine‐aminopeptidase in *S. droebachiensis* . No involvement in hydrogen networks was detected in aminopeptidase of *S. pallidus* .

## 4 Discussion

Ectothermic invertebrates, such as sea urchins, are highly impacted by increasing temperature, which exponentially affects the rates of all biochemical reactions and directly relates to higher energy demands (Clarke and Fraser 2004). Consequently, knowledge about the functional properties of enzymes that mediate most biochemical or metabolic processes at environmental temperatures is crucial. The efficiency and activity of enzymes at a given temperature are determined by their structural stability and flexibility. This, in turn, depends on the amino acid sequence and the resulting protein folding, which is mediated by interactions between amino acid residues (Pucci and Rooman 2017). Significant changes in enzyme efficiency or function can already be achieved by single or few mutations that lead to amino acid substitutions (Somero 2004). The occurrence of such convergent or parallel evolution across species is indicative of natural selection driven by environmental pressures (Rey et al. 2019).

We analyzed high‐quality transcriptomes from two sea urchin species from Subarctic and Arctic populations for CAAS. We found several CAAS between the two closely related *Strongylocentrotus* species and functional differences in enzyme activity and activation energy of several enzymes relevant in food utilization.

### 4.1 Genetic Variations Between Subarctic and Arctic Sea Urchins

Among the CAAS observed between Subarctic and Arctic transcriptomes, we detected higher numbers of hydrophilic residues in the Arctic populations. A higher number of hydrophilic surface residues is generally associated with cold adaptation (Wintrode and Arnold 2001). The same observations were made in the cold‐adapted endopeptidase trypsin by Schrøder Leiros et al. (2000). Additional patterns in amino acid composition, like the over‐ or underrepresentation of methionine, threonine, or arginine residues, are associated with cold adaptations, as varying frequencies of these amino acids were observed in psychrophilic enzymes compared to their thermophilic homologues (Saunders et al. 2003; Berthelot et al. 2019; Chao et al. 2020). A lower Arg/Lys ratio was additionally observed in cold‐adapted enzymes (Wintrode and Arnold 2001). While we did not find any overrepresented amino acids in the transcripts in which CAAS were detected, we calculated a slightly lower Arg/Lys ratio in the Arctic population. These compositional features support the structural flexibility of enzymes to ensure higher catalytic efficiencies (Hochachka and Somero 1984), indicating enzymatic cold adaptation in the Arctic population.

### 4.2 Amino Acid Substitutions

Lockwood and Somero (2012) proposed that some metabolic pathways and their enzymes might be more susceptible to environmental temperature changes and therefore may preferentially show temperature‐related adaptations. Frequently identified proteins and pathways relevant for thermal adaptation are involved in proteolysis, variation in heat tolerance (mainly heat shock proteins), and immune response (deMayo and Ragland 2025). We found in our study that while transcripts associated with all of these three categories express amino acid substitutions between the locations, they account only for a relatively small proportion of CAAS in both sea urchin species. However, since we focused on CAAS‐bearing transcripts, further thermal adaptations at the protein level outside the conserved regions investigated here, such as larger insertions or deletions, are not included in our findings.

The majority of CAAS in our data were found in transcripts associated with cellular and metabolic processes. This is in accordance with findings in freshwater snails exposed to chronic thermal stress (Mu et al. 2025) and genes affected in thermal adaptation in ectothermic vertebrates (Wollenberg Valero et al. 2014). Especially energy metabolism and digestive processes in sea urchins are of particular interest in this study, due to the ecological relevance of sea urchins as main grazers, and their consequently high biomass turnover (Hagen 1983; Fagerli et al. 2013; Filbee‐Dexter et al. 2020). Most CAAS associated with metabolic processes occurred in the lipid, protein, and carbohydrate metabolism. Sea urchins can digest all of these three major nutritive components with varying efficiencies (Obrietan et al. 1991; Hildebrand et al. 2023; Koch et al. 2025). We found amino acid substitutions between locations and in both species in TAG‐lipase, β‐galactosidase, and a metalloaminopeptidase, which was further identified through a BLASTX search as alanine‐aminopeptidase. These enzymes are present and active in the digestive tract of strongylocentroid sea urchins (Onitsuka et al. 2015; Koch et al. 2025). Substitutions in these individual enzymes are discussed below.

### 4.3 Molecular Structure and Functional Variation

Single amino acid changes between populations from different latitudes are often associated with thermal adaptations (e.g., Johns and Somero 2004; Foote et al. 2011). Similarly, certain enzymatic properties, such as flexibility of the molecule around the active site or lower activation energy (*E*_a_), are associated with optimized catalytic reactions at low temperatures (Åqvist et al. 2017; Somero 2003). However, to deriving the effect of single mutations on the structural and, more importantly, function remains a major challenge despite decisive computational progress (Bromberg and Rost 2009; Åqvist et al. 2017; Pan et al. 2022). Gleason (2019) proposed, therefore, to combine transcriptome analysis with phenotype studies to better relate genetic differences between populations with adaptations to local environmental conditions and probably reactions towards climate change. Consequently, we performed sequence analysis and measured functional properties of known digestive enzymes from the stomach and intestine of *S. droebachiensis* .

Among the biochemically analyzed enzymes, alanine‐aminopeptidase and β‐galactosidase showed CAAS and notable differences in biochemical properties between Subarctic and Arctic *S. droebachiensis* . Esterase and esterase/lipase, which may serve as proxies for lipid‐degrading enzymes, like TAG‐lipase (also containing one CAAS), were functionally assessed as well (Fojan et al. 2000).

Cold‐adapted enzymes typically show higher catalytic rates at low temperatures than their warm‐adapted orthologs (Clarke 1980; Schrøder Leiros et al. 2000; Somero 2003). Such adaptation can be beneficial in providing sufficient catalytic activity at low environmental temperatures. This was also evident in the investigated digestive enzymes from Subarctic and Arctic *S. droebachiensis* . Furthermore, we found significantly lower activation energies in the enzymatic reactions of the alanine‐aminopeptidase and esterase of the Arctic population of *S. droebachiensis* . Kinetic parameters like *E*_a_ are commonly used to investigate temperature adaptation of enzymes (Johnston and Walesby 1977; Fields and Houseman 2004). The lower amount of energy needed to facilitate catalytic reactions indicates higher enzymatic flexibility, easing the energetic demand for molecular confirmation changes during the reaction (Muffucci et al. 2020). Higher flexibility relates to changes in the amino acid sequence and the resulting modifications in interactions and forces between amino acid residues (Kokkinidis et al. 2012). Consequently, the significant changes in the *E*_a_ of the screened enzymes of Arctic sea urchins indicate thermal adaptations.

### 4.4 Consequences of Amino Acid Substitutions on Biochemical Properties

A serine‐to‐threonine substitution (position 201–213) was identified near the predicted catalytic triad within the TAG‐lipase of Subarctic and Arctic populations (Gatti‐Lafranconi et al. 2008; Rivera‐Pérez et al. 2011). Similar mutations have been experimentally linked to cold adaptation in bacterial lipases (Sharma et al. 2017), where the occurrence of threonine shifted the thermal optimum towards lower temperatures. Sharma et al. (2017) concluded that the higher hydrophilicity of threonine, as well as a loss in one hydrogen bond due to changes in molecule structure, might be responsible for the strong effect of this mutation. This seems likely, as hydrogen bonds are significant factors determining enzyme stability (Hubbard and Kamran Haider 2010). We observed a similar mutation, though at a different position in TAG‐lipase, which caused no change in the amount of hydrogen bonds at the CAAS site. However, the distance of the hydrogen bond between H188 and T213 in the Arctic enzyme was slightly larger than the distance between H176 and S201 in the Subarctic enzyme. A greater hydrogen bond distance correlates with reduced binding strength (Herschlag and Pinney 2018) and increased flexibility of the enzyme (Dai et al. 2019). Therefore, we conclude that the occurrence of serine in the Subarctic and threonine in the Arctic enzyme contributes to cold adaptation.

Psychrophilic enzymes have fewer stabilizing hydrogen bonds, promoting structural flexibility (Vogt et al. 1997; Kim et al. 1999; D'Amico et al. 2002). While we noted lower numbers of hydrogen bonds and hydrogen bond networks in Arctic TAG‐lipase, no corresponding observations were made in Subarctic and Arctic alanine‐aminopeptidase and β‐galactosidase.

Adaptations towards low temperatures of the carbohydrate degrading enzyme β‐galactosidase have been reported in several studies, and mostly in procaryotic enzymes (Asraf and Gunasekaran 2010; Kumar et al. 2014; Karan et al. 2020). A reduction of isoleucine was found in thermophilic microbial β‐galactosidase (Kumar et al. 2014). In previous studies, the substitution of isoleucine by the more hydrophilic valine in the hydrophobic core of trypsin was attributed to lower enzyme stability and therefore would be beneficial in cold adaptation (Schrøder Leiros et al. 2000). In contrast, our study found that the Arctic β‐galactosidase contains isoleucine at the substitution site, which was exchanged for valine in the Subarctic enzyme and positioned more towards the periphery of the enzyme. DasSamara et al. (2013) found that the nonpolar amino acids valine, isoleucine, and leucine are often substituted for each other in cold‐adapted Archaean enzymes. However, the effects on the molecular structure, and therefore catalytic activity, are unknown.

Further computational methods, such as the calculation of the free energy ΔΔ*G*, can predict functional changes caused by specific mutations. ΔΔ*G* is a measure of the change in Gibbs free energy (Δ*G*) resulting from a mutation. It indicates a change in the stability of an enzyme due to different forces acting between neighboring amino acids (Pires et al. 2014). We simulated point mutations at CAAS sites in digestive enzymes by replacing the Arctic amino acid with the amino acid found in the Subarctic population and vice versa. We expected negative ΔΔ*G*, indicating destabilizing effects, in the Subarctic enzymes, and the opposite in Arctic enzymes. However, all simulated mutations, regardless of Arctic or Subarctic enzyme, resulted in destabilizing effects. Nevertheless, in the case of TAG‐lipase and β‐galactosidase, the destabilizing effects were greater when the mutation was simulated in the Arctic population. We conclude that the Subarctic enzymes are less affected by the mutation than their Arctic counterparts. These findings suggest that the enzymes in the Arctic populations are less stable and more flexible, which may enhance catalytic activity at low temperatures.

In summary, we provide evidence for genetic variation between Arctic and Subarctic sea urchins, suggesting adaptation towards the local environmental conditions. Especially in TAG‐lipase and β‐galactosidase substitutions and molecular structures point towards cold adaptation of these enzymes. However, the functional biochemical differences observed in alanine‐aminopeptidase could not be related to sequence variation.

### 4.5 Adaptation of Sea Urchins in the Face of Ecosystem Change

As ecosystems change rapidly due to global warming, the understanding of the potential of species to cope with environmental shifts is gaining importance, particularly with regard to temperatures. Although it is challenging to predict adaptive potentials towards future changes in ecosystems (Urban et al. 2024), valuable insights can be obtained by comparing physiological and genetic properties among populations of related taxa or even the same species inhabiting different environments (e.g., Halbritter et al. 2015; Rose et al. 2018; Choquet et al. 2023). Consequently, sampling across a latitudinal gradient can help to identify local temperature adaptations (De Frenne et al. 2013; Pespeni and Palumbi 2013). Our findings are supported by studies on the genetic variation of the related purple sea urchin *S. purpuratus* (Pespeni and Palumbi 2013; Petak et al. 2023). The authors showed adaptations with regard to temperature and pH and concluded high adaptive potential towards environmental changes.

Physiological and biochemical adaptations affect the grazing activity of sea urchins in kelp‐dominated habitats. As sea urchins are the main grazers on benthic macroalgae, our analysis focused on the digestive enzymes involved in the breakdown of algal components (Koch et al. 2025). Our results indicate local adaptations to improve metabolic food utilization and energy‐related pathways. Since an organism's energy budget is tightly linked to temperature (Sokolova et al. 2012), adaptations that optimize digestion at lower temperatures may be particularly beneficial in Arctic populations. In Subarctic *S. droebachiensis* , grazing rates increase with temperature up to 10°C. Above this threshold, grazing activity gradually decreases. A similar experiment with Arctic *S. droebachiensis* revealed an increase in grazing rates up to 6°C (M. Koch, personal communication). These maximal grazing activities coincided with the respective summer temperatures at either location (Richard et al. 2012; Aliani et al. 2016; Noufal et al. 2017; Koch et al. 2024) and suggest a plastic adjustment of feeding and energy uptake towards local environmental temperatures. Despite the limited sample size in our biochemical study, we found significant differences in enzyme activity and *E*_a_ in several enzymes. These results support our conclusion that further rising temperatures in the next decades could lead to elevated metabolic rates and therefore higher energy requirements, especially in Arctic sea urchins. This, in turn, can result in higher grazing activities of Arctic sea urchins with detrimental effects on local kelp communities with cascading effects on kelp‐ecosystem functions and biodiversity (Carranza et al. 2024).

## 5 Conclusion

We found genetic and functional variations in Subarctic and Arctic sea urchins. Enzyme activities suggest generally higher catalytic activity and therefore metabolic efficiency at lower temperatures in the Arctic populations, indicating an adaptive reaction towards local temperature regimes. Molecular substitutions in several transcript groups suggest that the enzymatic differences between the populations have underlying genetic causes, indicating permanent adaptation rather than reversible acclimation of metabolic processes. These latitudinal differentiations occurring in both species seem to correspond with the local ecosystem and temperature regime. The high genetic diversity of *Strongylocentrotus* species from the Arctic and Subarctic is a strong indication for successful responses towards changing environmental conditions in the past. Potential adaptation towards ongoing environmental changes, particularly temperature, seems possible therefore, but will also depend on the high rate of these abiotic changes.

## Author Contributions

**Marie Koch:** conceptualization (lead), data curation (lead), formal analysis (lead), investigation (lead), methodology (lead), validation (lead), visualization (lead), writing – original draft (lead). **Sylke Wohlrab:** formal analysis (supporting), methodology (supporting), writing – review and editing (supporting). **Lars Harms:** methodology (supporting), writing – review and editing (supporting). **Reinhard Saborowski:** funding acquisition (lead), project administration (lead), supervision (lead), writing – review and editing (lead).

## Conflicts of Interest

The authors declare no conflicts of interest.

## Supporting information


**Table S1:** Overview of identified and functionally annotated CAAS.
**Table S2:** List of all amino acid sequences of TAG‐lipase used for structure analysis.
**Table S3:** List of all amino acid sequences of β‐galactosidase used for structure analysis.
**Table S4:** List of all amino acid sequences of alanine‐aminopeptidase (metalloaminopeptidase) used for structure analysis.
**Table S5:** Enzyme activity data.

## Data Availability

Enzyme activity data (Table S5) as well as a table containing all identified and functionally annotated CAAS (Table S1) are listed in the Supporting Information. Amino acid sequences of TAG‐lipase, alanine‐aminopeptidase, and β‐galactosidase used for structure analysis are listed in Tables S2, S3 and S4. The sequence data of the transcriptomes are available as BioProjects in the NCBI database: *S. droebachiensis* Arctic: PRJNA1307582, Subarctic: PRJNA1307474; *S. pallidus* Arctic: PRJNA1308330, Subarctic: PRJNA1307779; *S. purpuratus* : PRJNA1308562.
